# Infiltration-RNAseq: transcriptome profiling of Agrobacterium-mediated infiltration of transcription factors to discover gene function and expression networks in plants

**DOI:** 10.1186/s13007-016-0141-7

**Published:** 2016-10-19

**Authors:** Donna M. Bond, Nick W. Albert, Robyn H. Lee, Gareth B. Gillard, Chris M. Brown, Roger P. Hellens, Richard C. Macknight

**Affiliations:** 1 Department of Biochemistry, University of Otago, PO Box 56, Dunedin, 9054 New Zealand; 2 The New Zealand Institute for Plant and Food Research Limited, Private Bag 11-600, Palmerston North, New Zealand; 3 Centre for Tropical Crops and Biocommodities, Institute for Future Environments, Queensland University of Technology, GPO Box 2434, Brisbane, QLD 4001 Australia; 4 Department of Chemistry, Biotechnology and Food Science, Norwegian University of Life Sciences, 1432 Ås, Norway

**Keywords:** Transcription factor, *Agrobacterium tumefaciens*, Infiltration, *Medicago truncatula*, MtLAP1, Anthocyanin, RNAseq, Transcriptome, *Nicotiana benthamiana*

## Abstract

**Background:**

Transcription factors (TFs) coordinate precise gene expression patterns that give rise to distinct phenotypic outputs. The identification of genes and transcriptional networks regulated by a TF often requires stable transformation and expression changes in plant cells. However, the production of stable transformants can be slow and laborious with no guarantee of success. Furthermore, transgenic plants overexpressing a TF of interest can present pleiotropic phenotypes and/or result in a high number of indirect gene expression changes. Therefore, fast, efficient, high-throughput methods for assaying TF function are needed.

**Results:**

Agroinfiltration is a simple plant biology method that allows transient gene expression. It is a rapid and powerful tool for the functional characterisation of TF genes *in planta*. High throughput RNA sequencing is now a widely used method for analysing gene expression profiles (transcriptomes). By coupling TF agroinfiltration with RNA sequencing (named here as Infiltration-RNAseq), gene expression networks and gene function can be identified within a few weeks rather than many months. As a proof of concept, we agroinfiltrated *Medicago truncatula* leaves with *M. truncatula LEGUME ANTHOCYANIN PRODUCITION 1* (*MtLAP1*), a MYB transcription factor involved in the regulation of the anthocyanin pathway, and assessed the resulting transcriptome. Leaves infiltrated with *MtLAP1* turned red indicating the production of anthocyanin pigment. Consistent with this, genes encoding enzymes in the anthocyanin biosynthetic pathway, and known transcriptional activators and repressors of the anthocyanin biosynthetic pathway, were upregulated. A novel observation was the induction of a R3-MYB transcriptional repressor that likely provides transcriptional feedback inhibition to prevent the deleterious effects of excess anthocyanins on photosynthesis.

**Conclusions:**

Infiltration-RNAseq is a fast and convenient method for profiling TF-mediated gene expression changes. We utilised this method to identify TF-mediated transcriptional changes and TF target genes in *M. truncatula* and *Nicotiana benthamiana*. This included the identification of target genes of a TF not normally expressed in leaves, and targets of TFs from other plant species. Infiltration-RNAseq can be easily adapted to other plant species where agroinfiltration protocols have been optimised. The ability to identify downstream genes, including positive and negative transcriptional regulators, will result in a greater understanding of TF function.

**Electronic supplementary material:**

The online version of this article (doi:10.1186/s13007-016-0141-7) contains supplementary material, which is available to authorized users.

## Background

Transcription factors (TFs) play a major role in plant development and their response to the environment. An understanding of the function(s) of TFs has been elucidated by a number of reverse genetic tools, including gene knockout and gene overexpression strategies. As some TFs display unique properties, the overexpression strategy has been particularly effective in revealing TF function [1–3]. However, these methods rely on stable transformation of plant cells, but obtaining stable transformants of crop plants is a time-consuming and labour-intensive process that is often inefficient.

In contrast to making stable transgenics, *Agrobacterium*-mediated transient expression of genes provides a rapid and simple alternative for analysing gene function [4]. The most commonly used transient expression method involves ‘agroinfiltration’—injection of an *Agrobacterium* suspension (harbouring a gene of interest on a T-DNA vector) through the stomata and into the mesophyll of expanded leaves using a needleless syringe. Copies of the T-DNA are transferred from the *Agrobacterium* into the leaf parenchyma cells. Although only a low number of T-DNA copies integrate into the plant chromosomes, the non-integrated T-DNAs are transiently expressed for several days [5].

Leaves of *Nicotiana benthamiana* or *Nicotiana tabacum* have proven to be a reliable system for agroinfiltration, where a large fraction of cells are transformed, and in extreme cases can result in 50 % of the total soluble leaf protein encoded by the transferred gene [6]. This has led to applications where pharmaceutically active proteins are produced via agroinfiltration at a commercially viable scale [7]. In addition to bulk protein production, agroinfiltration has been used to address a range of biological questions. For example, it provides an in vivo system to identify protein-protein interactions [8], examine protein localization [9], study host-pathogen interactions [10] and understand TFs that regulate specific promoter sequences [11–13] and specific gene expression profiles [14]. Detailed aspects of gene regulation, such as the role of upstream open reading frames (uORFs), introns within 5′-untranslated regions (5′UTRs) and non-canonical translation initiation have also been investigated via agroinfiltration [15–17]. The key advantage of agroinfiltration over stable transgenics is that experimental results can be generated after a few days, rather than many months, and compared to using stably transformed plant lines, transient expression assays eliminate variation due to different chromosomal positions and epigenetic states of the transformed constructs.

Although leaves of *Nicotiana* species are widely used for agroinfiltration, other plants have been used with variable success [18–24]. We have recently established a robust method for agroinfiltration of *Medicago truncatula* [25], an important model legume that is used extensively by researchers throughout the world. Adapting the agroinfiltration method for use in *Medicago* provides a more relevant tool for researchers of legume species, as the production of stably transformed leguminous plants is difficult and time consuming. A powerful use of the transient assay system in this species is the identification of direct targets of a specific TF [25]. For example, gene expression analysis via quantitative real-time PCR demonstrated that candidate downstream targets of *M. truncatula* LEGUME ANTHOCYANIN PRODUCITON 1 (MtLAP1), a MYB TF involved in the regulation of the anthocyanin biosynthesis pathway [26], were upregulated in leaves that were agroinfiltrated with *35S:MtLAP1* [25].

Constitutive expression of *MtLAP1* in transgenic or agroinfiltrated *M. truncatula* induces massive accumulation of anthocyanin pigments [25, 26]. Microarray analysis of transgenic *Medicago* plants over-expressing *MtLAP1* found over 70 genes were up-regulated, many of which were involved in anthocyanin biosynthesis [26]. However, in addition to the time consuming nature of producing transgenic plants, stable overexpression of TFs can result in a high number of indirect gene expression changes being called, and pleiotropic phenotypes that need to be interpreted with caution [1–3]. We propose a more simple experiment to understand TF-mediated gene expression changes that does not rely on the production of stable transformants—agroinfiltration of a TF followed by high-throughput RNA sequencing. Due to the short time frame of this type of experiment, we speculate that the gene expression analysis will be less noisy and allow greater discovery of specific downstream genes and thus gene expression networks.

In this article, we present the method of ‘Infiltration-RNAseq’—a simple and rapid analysis of gene expression profiles that allows the discovery of novel downstream targets of a chosen TF. In order to establish this technique, RNA sequencing analysis was performed on *M. truncatula* leaves agroinfiltrated with *35S:MtLAP1*, the primary TF involved in the anthocyanin biosynthetic pathway [25, 26]. Infiltration-RNAseq was further validated using *Nicotiana benthamaina* leaves to determine the transcriptional changes resulting from infiltration of *Agrobacterium* either alone or expressing TFs from another plant or not normally expressed in leaves.

## Results

### Infiltration-RNAseq: for discovery of downstream targets of transcription factors

**Fig. 1:**
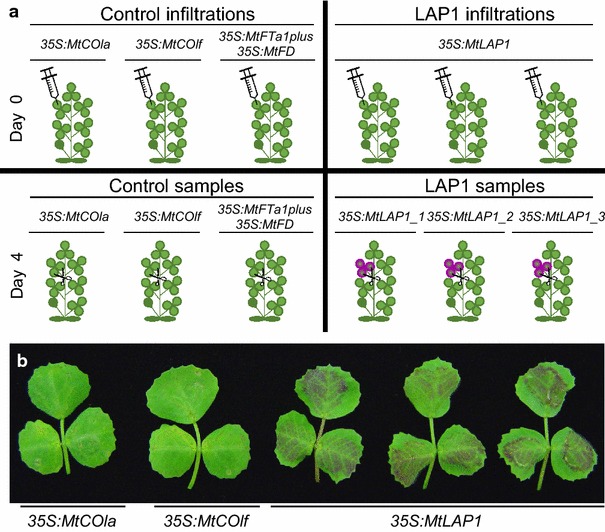
Agroinfiltration of *35S:MtLAP1* induces anthocyanin pigment production in *Medicago truncatula* leaves. **a** Experimental design of *M. truncatula MtLAP1* Infiltration-RNAseq experiment. On Day 0 (*top*) all three leaflets of the 4th trifoliate leaf of healthy 3 weeks old plants were agroinfiltrated (represented by syringe) with ‘Control’ infiltrations (*left* hand side) or ‘LAP1’ infiltrations (*right* hand side). For the Control infiltrations, a single plant was infiltrated with either: *35S:MtCOla*, *35S:MtCOlf* or *35S:MtFTa1plusMtFD* [56, 57]. For the LAP1 infiltrations, a triplicate set of plants were infiltrated with *35S: MtLAP1* [25]. Four days post infiltration (*bottom*), all three leaflets of agroinfiltrated leaves were harvested (represented by scissors) in preparation for RNA extraction and analysis. The resulting control samples were: 35S:MtCOla, 35S: MtCOlf and MtFTa1plusMtFD, and the resulting LAP1 samples (*purple* leaves due to anthocyanin production) were 35S: MtLAP1_1, 35S: MtLAP1_2 and 35S: MtLAP1_3. **b** All three leaflets of the 4th trifoliate leaf of healthy 3 weeks old plants were agroinfiltrated with *35S:MtLAP1*, *35S:MtCOla* or *35S:MtCOlf*. The *35S:MtCOla* and *35S:MtCOlf* constructs overexpress *Medicago* genes that are not involved in anthocyanin biosynthesis [56, 57] (see “Methods”). Agroinfiltrated leaves were harvested for photographing 4 days post infiltration. *Note*: these leaves are representative agroinfiltrations and were not used for RNA extraction and downstream analyses

The efficiency and versatility of the agroinfiltration technique in *M. truncatula* [25] prompted us to test the possibility of combining agroinfiltration with next-generation RNA sequencing as a rapid method to discover novel downstream targets of a TF. As a proof of concept, we agroinfiltrated *MtLAP1*, a MYB TF that regulates anthocyanin biosynthesis, into leaves of healthy 3-week-old *M. truncatula* plants (see “Methods”). A number of control constructs (Fig. 1a) that overexpress different *Medicago* genes not involved in anthocyanin biosynthesis were also agroinfiltrated into *Medicago* leaves. This type of experimental control removes the effects of agroinfiltration i.e. genes involved in the pathogen disease response will be masked as they will be similarly expressed in leaves infiltrated with the control or test constructs.

Infiltration of *Agrobacterium tumefaciens* containing the *35S:MtLAP1* construct results in high *MtLAP1* expression in *M. truncatula* [25]. *Medicago* leaves overexpressing the MtLAP1 TF turn red (Fig. 1b; [25, 26]), indicating the anthocyanin biosynthetic pathway is upregulated. Leaves from plants agroinfiltrated with *35S:MtLAP1* or control constructs were harvested 4 days post-infiltration (Fig. 1a; “Methods”). While high *MtLAP1* expression was observed 3 days post infiltration [25], we wanted to capture the highest gene expression changes of MtLAP1 target genes. Therefore, we reasoned that maximal translation of *MtLAP1* mRNA and thus function of MtLAP1 protein would occur about a day later. As with infiltration of *N. benthamiana*, it is not recommended to sample more than 5 days post-infiltration as RNA silencing mechanisms will act on the agroinfiltrated construct and result in low reproducibility between samples and experiments [12, 27]. RNAseq libraries were made with RNA from the different leaf samples and the resulting RNAseq data was analysed to determine the gene expression changes (see “Methods”). We have named this method of gene expression analysis and discovery ‘Infiltration-RNAseq’.

### Transcriptional regulation of anthocyanin biosynthetic genes in *Medicago truncatula* leaves via agroinfiltration of *35S:MtLAP1*

**Fig. 2:**
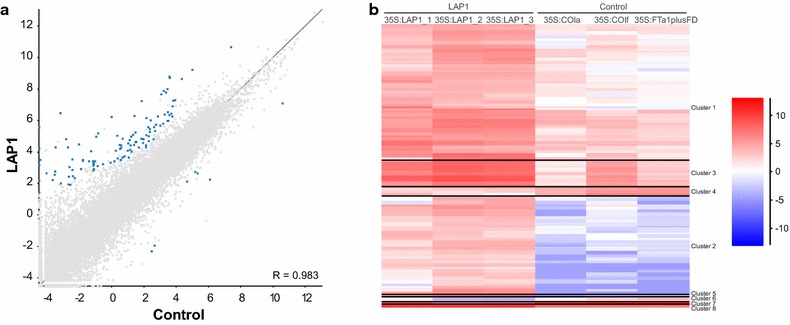
Significantly differentially expressed genes between ‘Control’ and ‘LAP1’ agroinfiltration conditions. **a** *Scatter plot* showing the correlation for significantly differentially expressed genes between Control (x-axis) and LAP1 (y-axis) replicate sets (Pearson correlation value = 0.983). Differential expression analysis between the two replicate sets was performed on raw counts with annotated mRNAs via the DESeq2 Filter [35] and combined with the Intensity Difference Filter using SeqMonk [62] (see “Methods”). mRNAs were considered significantly differentially expressed when the adjusted *P* value was <0.05 (*blue dots*). *Grey dots* represent all other mRNAs in the *M. truncatula* genome (version Mt4.0), showing the relationship between the quantitated values in the Control and LAP1 replicate sets. **b** Hierarchical clustering of differentially expressed genes between ‘Control’ and ‘LAP1’ agroinfiltration conditions. With a correlation coefficient of 0.7, 109 genes fell in to 8 clusters with three major categories: low-moderate expression in control conditions, moderate induction by *MtLAP1* infiltration (cluster 1); very low expression in control conditions, induced by *MtLAP1* infiltration (cluster 2); moderate expression in control conditions, highly induced in response to *MtLAP1* infiltration (cluster 3). Values are log_2_-transformed library-normalized/median-normalized counts

To determine the gene expression profile established by *MtLAP1* overexpression, we analysed transcripts from leaves agroinfiltrated with *35S:MtLAP1* or control constructs using strand-specific RNAseq. Using stringent parameters (see “Methods”), we identified 118 significantly differentially expressed genes between ‘Control’ and ‘LAP1’ agroinfiltrations (*P* value <0.05) (Fig. 2a; Additional file 1: Table S1). Of the significantly differentially expressed genes (*P* value <0.05), 111 were upregulated (including *MtLAP1*, which displayed a ≥1000-fold induction) and 7 were downregulated suggesting that *MtLAP1* primarily acts as a transcriptional activator. Unless otherwise stated, when the fold-change in expression is given it refers to genes determined to be significantly differentially expressed (*P* value <0.05) (Fig. 2; Additional file 1: Table S1). The transcripts displaying significant differential upregulation clustered into three major groups with distinct gene expression profiles (Fig. 2b; Additional file 2: Table S2, Additional file 3: Table S3 and Additional file 4: Table S4)—cluster 1 represents genes that were low-moderately expressed in control conditions and moderately induced by *MtLAP1* infiltration; cluster 2 represents genes that were not or very lowly expressed in control samples and induced by *MtLAP1* infiltration; cluster 3 represents genes that were moderately expressed in control conditions and highly expressed in response to *MtLAP1* infiltration.

**Fig. 3:**
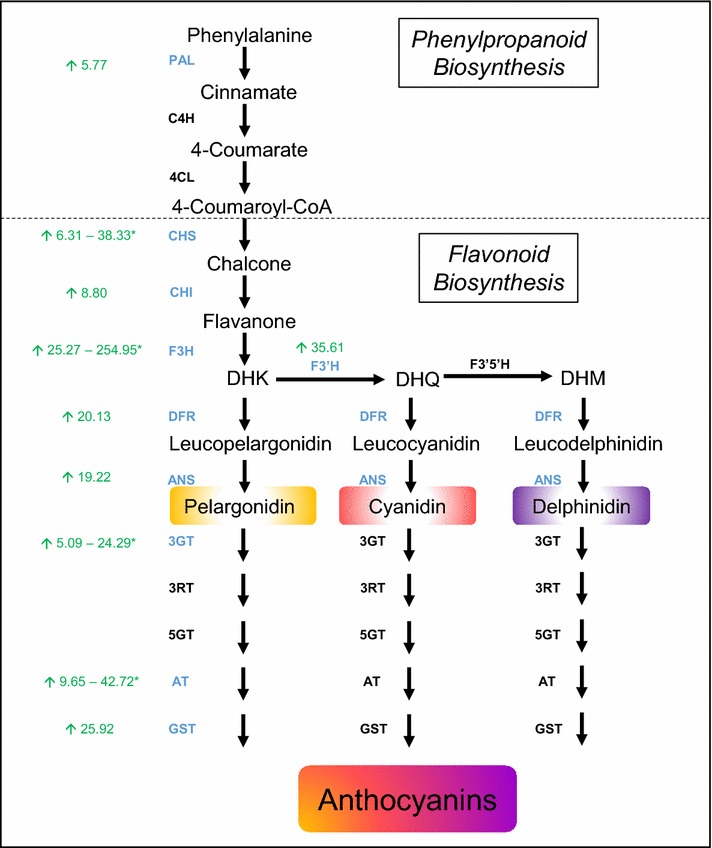
The core pathway for anthocyanin biosynthesis. The general phenylpropanoid pathway is catalysed by phenylalanine ammonia lyase (PAL), cinnamate 4-hydroxylase (C4H) and 4-coumaroyl CoA ligase (4CL). Enzymes involved in flavonoid biosynthesis are chalcone synthase (CHS), chalcone isomerase (CHI), flavanone 3-hydroxylase (F3H), flavanone 3′-hydroxylase (F3′H) and flavanone 3′5′-hydroxylase (F3′5′H), which produce dihydroflavanols: dihydrokaempferol (DHK), dihydroquercetin (DHQ) and dihydromyricetin (DHM), respectively. Anthocyanins are synthesized by dihydroflavanol 4-reductase (DFR) and anthocyanin synthase (ANS), and stabilised by 3-glucosyl-transferase (3GT), 3-rhamnosyl transferase (3RT), 5-glucosyl transferase (5GT) and anthocyanin acyl transferase (AT). Biosynthetic genes identified as being significantly differentially expressed (*P* value <0.05) are in *blue*; numbers in *green* are the fold change of expression observed for each differentially expressed gene between Control and LAP1 conditions (Additional file 1: Table S1); *green upward arrow* represents upregulation; *Asterisk* multiple isoforms of genes encoding these enzymes are upregulated

Genes encoding most of the known enzymes in the flavonoid pathway that produce anthocyanidins (such as pelargonidin, cyanidin and delphinidin) were upregulated in response to *MtLAP1* overexpression (Fig. 3; Additional file 1: Table S1). This is consistent with the fact that leaves agroinfiltrated with *MtLAP1* displayed a red phenotype (Fig. 1b; [25]), indicative of anthocyanin pigment production. Interestingly, the majority of the anthocyanin biosynthetic genes were part of cluster 1 and 3—genes which are low-moderately expressed in control conditions and moderately-highly induced in response to *MtLAP1* infiltration (Additional file 2: Table S2 and Additional file 4: Table S4). The low-moderate level of expression of these genes in control conditions likely occurs because other flavonoids accumulate in *M. truncatula* leaves. These include flavonols, flavones, isoflavonoids [28], proanthocyanidins (restricted to trichomes) [29] and subtle anthocyanin leaf markings [30]. Flavonoid 3′5′-hydroxylase (F3′5′H) was the only gene from the flavonoid biosynthesis branch of the anthocyanin pathway that was not upregulated, and showed no or very low expression in control samples. This suggests that the anthocyanidin conjugates in *MtLAP1* agroinfiltrated leaves are unlikely to be delphinidin derivatives. Consistent with this, delphinidin derivatives were not identified when extracts from transgenic *Medicago* overexpressing *MtLAP1* were assessed by liquid chromatography-tandem mass spectrometry (LC-MS/MS) [21], although delphinidin derived proanthocyanidins (gallocatechin) are produced in *Medicago* [31]. This could be due to the inability of MtLAP1 to induce *F3*′*5*′*H* expression, which may instead be regulated by the proanthocyanindin MYB gene (*MYB14*) [31] to produce delphinidin-based proanthocyanidins.

Diverse and stable anthocyanin pigments are derived from anthocyanidins that are immediately modified by glycosylation, acylation, and/or methylation [32]. Overexpression of *MtLAP1* induced a number of glucosyltransferases and malonyltransferases (anthocyanin acyl transferases), and a glutathione-*S*-transferase (Additional file 1: Table S1; [26]) that are likely to be responsible for these modifications. Modification of the anthocyanidin compounds is essential for their transport into the vacuole where the water-soluble anthocyanin pigments are stored. Transport of diverse flavonoid compounds into the vacuole involves Multidrug and Toxic compound Extrusion (MATE) proteins [33, 34]. Agroinfiltration of *MtLAP1* into *Medicago* leaves resulted in a significant 10-fold induction of a MATE transporter (*Medtr1g021740*) (Additional file 1: Table S1). This MATE transporter has not yet been characterised, but it is distinct from MtMATE1 and MtMATE2, which transport proanthocyanidin monomers and various flavonoid-/anthocyanin-glycosides, respectively [33, 34]. Differential expression analysis via DESeq2 [35] identified MtMATE2 (*Medtr1g100180*) to be up-regulated 29-fold upon *MtLAP1* infiltration (Additional file 5: Figure S1), however MATE2 did not meet the criteria for the stringent Intensity Difference Filter (*P* value >0.05), most probably due to high variation in expression between the biological replicates for the different conditions.

### MtLAP1-regulated transcriptional activators and repressors controlling anthocyanin pigmentation in *Medicago truncatula*

**Fig. 4:**
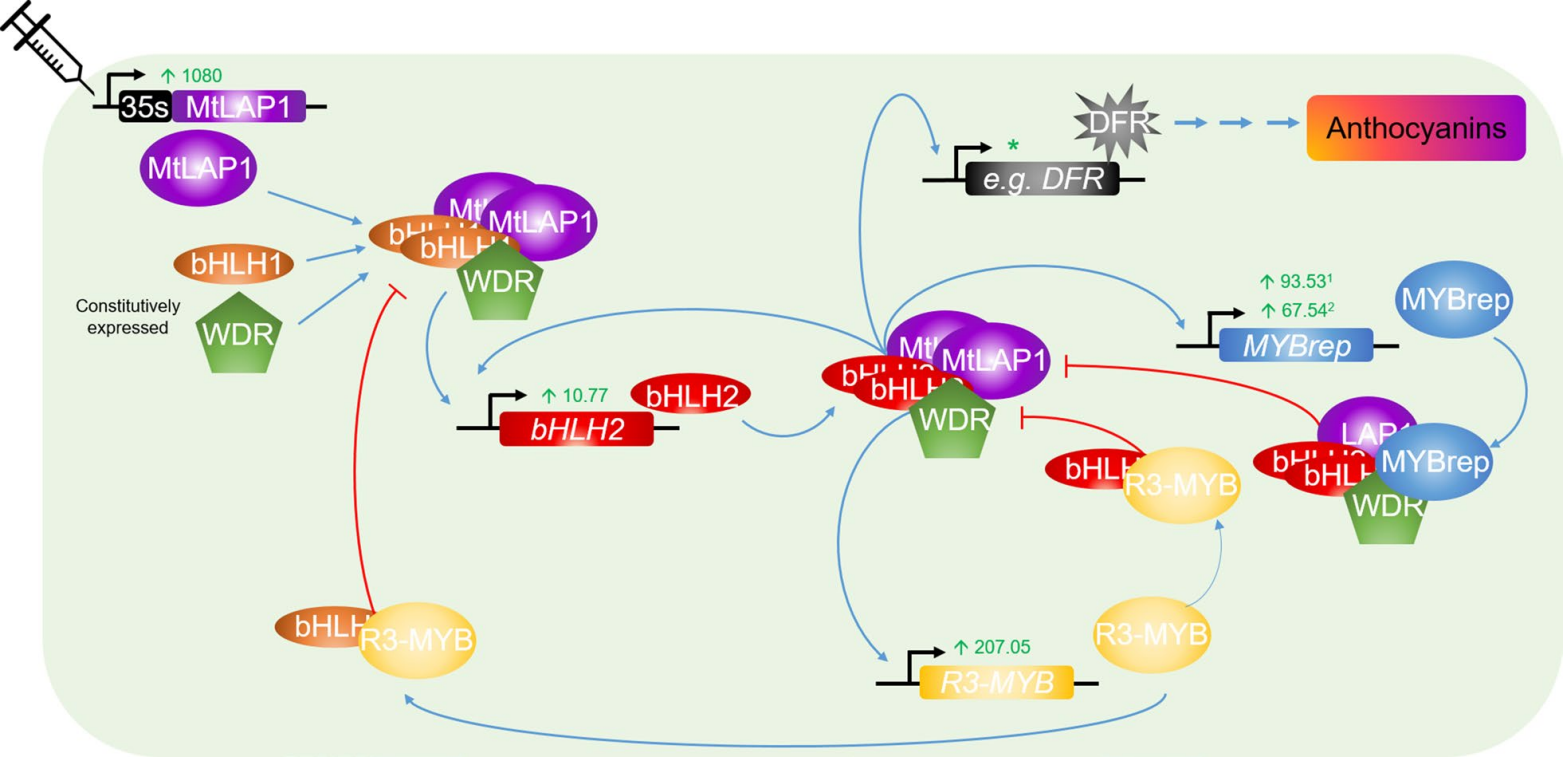
Agroinfiltration of *MtLAP1* initiates the anthocyanin gene regulatory network in *M. truncatula*: proposed model. Upon agroinfiltration of *35S:MtLAP1*, MtLAP1 can form an MBW activation complex with constitutively expressed bHLH1 (basic helix-loop-helix clade 1; *Medtr8g098275*) and WDR (WD-repeat proteins; *Medtr3g192840* and/or *Medtr7g084810*) proteins, which activates expression of bHLH2 (basic helix-loop-helix clade 2; *Medtr1g072320*; MtTT8). A core MBW complex, containing bHLH2, forms that positively autoregulates bHLH2 expression and activates expression of the anthocyanin biosynthetic genes (represented by DFR; dihydroflavanol 4-reductase) resulting in anthocyanin pigment production. The core MBW also activates expression of *MYBrep* genes (R2R3-MYB; *Medtr4g585530*/MtMYB530 and *Medtr5g079670*/MtMYB2) and R3-MYB (*Medtr2g088730*/MtMYB730). The inclusion of the MYB repressor into the MBW complex results in the transcriptional repression of target promoters of the core MBW complex (*bHLH2*, *MYBrep* and *R3*-*MYB*). Feedback inhibition is provided by R3-MYB by inhibiting the formation of new MBW complexes by titrating bHLH1 and bHLH2. Numbers in *green* are the fold change of expression for each gene significantly differentially expressed (*P* value <0.05; *MtLAP1*, bHLH2 = *Medtr1g072320*/MtTT8; MYBrep = *Medtr4g485530*; R3-MYB = *Medtr2g088730*/MtMYB730); *upward arrow* represents upregulation; ^1^ *Medtr4g585530*/MtMYB530 MYBrep; ^2^ *Medtr5g079670*/MtMYB2 MYBrep; *Asterisk* See Fig. 3 for fold change expression of biosynthetic genes

Anthocyanin biosynthesis is regulated primarily at the transcriptional level by a set of conserved TFs [36, 37]. These include R2R3-MYB proteins (such as MtLAP1), basic helix-loop-helix (bHLH) proteins and WD-repeat (WDR) proteins, which can form a complex (MBW complex) that activates the biosynthetic genes for anthocyanin synthesis. The R2R3-MYB proteins that activate anthocyanin synthesis are typically present in gene families, with individual members controlling specific pigmentation patterns throughout the plant, and function with common bHLH and WDR factors [36]. In our Infiltration-RNAseq experiment, the *Medicago* bHLH1 gene (*Medtr8g098275*) and WDR genes (*Medtr3g192840* and *Medtr7g084810*) that are similar to the AtEGL3 clade bHLH and AtTTG1, respectively, were present in both Control and LAP1 conditions suggesting that they are constitutively expressed (Fig. 4; Additional file 1: Table S1). Upon agroinfiltration of *35S:MtLAP1*, the MtLAP1 protein will be available to complex with bHLH1 and WDR forming a transcriptional activation complex [36, 37].

Known targets of the MYB transcriptional activation complex for anthocyanin biosynthesis include *bHLH2* (an AtTT8 clade bHLH). A core MBW transcriptional activation complex containing bHLH2 forms, which activates the expression of anthocyanin biosynthesis genes and reinforces expression of itself [37]. Upon *MtLAP1* agroinfiltration, the *M. truncatula* *bHLH2* gene, *Medtr1g072320/MtTT8* [38], displayed a tenfold induction (Fig. 4; Additional file 1: Table S1), suggesting the resulting bHLH2 protein could become part of the core MBW complex. This complex can also activate the expression of the R2R3-MYB repressors [37, 39, 40]. We found two R2R3-MYB repressor genes that were upregulated by *MtLAP1* agroinfiltration: *Medtr4g485530/MtMYB530*, homologous to *TrMYB133*, displayed a 93-fold induction, and *Medtr5g079670/MtMYB2*, homologous to *TrMYB134*, displayed a 67-fold induction [40, 41] (Fig. 4; Additional file 1: Table S1). The inclusion of the R2R3-MYB repressor into the core MBW complex results in active transcriptional repression of target promoters of the core MBW complex [37]. Similarly, the core MBW complex activates the expression of a smaller R3-MYB repressor, which provides feedback inhibition by inhibiting the formation of new core MBW complexes by titrating bHLH factors [37, 39]. The *Medicago* R3-MYB likely to be involved in this process is *Medtr2g088730/MtMYB730* [41], which displayed a 207-fold induction in *MtLAP1* agroinfiltrated leaves (Fig. 4; Additional file 1: Table S1). The deduced amino acid sequence of this gene contains the bHLH-interacting motif [42], which is highly predictive for binding anthocyanin-related bHLH partners, and is phylogenetically related to characterised R3-MYB proteins that regulate anthocyanin/proanthocyanidin synthesis (Additional file 6: Figure S2). This suggests feedback repression for anthocyanin synthesis by R3-MYBs occurs in leguminous species.

Our Infiltration-RNAseq experiment was successful in identifying potential positive and negative regulators of anthocyanin biosysnthesis in *M. truncatula*. Importantly, all of the genes encoding the positive and negative transcriptional regulators of the anthocyanin pathway were present in cluster 2 (Fig. 2b; Additional file 3: Table S3), which includes genes that had little or no expression in control samples, but were greatly induced upon *MtLAP1* agroinfiltration. This pattern of gene expression is consistent with the induction of visible anthocyanin pigmentation upon infiltration with *MtLAP1* (Fig. 1b), as expected for a probable transcriptional activator. The positive and negative transcriptional regulators of this system, especially the R3-MYB repressor, act to provide homeostasis for the cell—in leaves, anthocyanins can provide photo-protection from excess light, but excessive anthocyanin accumulation can compromise photosynthesis [43].

### Other genes regulated by *MtLAP1* overexpression

Gene ontology enrichments [44] for the remaining significantly differentially expressed genes (Additional file 1: Table S1) primarily identified the following processes: carbohydrate metabolism; hydrolase activity; acting on glycosyl bonds. Anthocyanin molecules are decorated with sugar molecules (such as glucose, rhamnose, galactose, arabinose and xylose), which act to stabilise the anthocyanins [32]. We speculate that *MtLAP1*-induced genes encoding enzymes involved in hydrolysis of glycosidic bonds between two or more carbohydrates could result in the supply of sugar molecules for the glycosylation process.

Homologues of MtLAP1 (AtMYB75/AtPAP1) have been shown to act as a repressor of lignin biosynthetic genes and secondary cell-wall metabolism genes [45–47]. Altered expression of *MYB75/PAP1* results in a major shift of the carbon partitioning to major sinks [47]. The *Arabidopsis myb75*-*1* mutant has an increase in carbon accumulation of monolignols, precursors of lignin in secondary cell walls, which results in increased cell wall thickness. In contrast, a gain-of-function mutation of *MYB75* results in an increase in carbon accumulation in anthocyanins. A possible source of the carbon needed to produce the increased number of anthocyanin pigments in response to the overexpression of *MtLAP1* is the plant cell wall. *MtLAP1* agroinfiltration induced the expression of a number of genes encoding enzymes associated with, or the breakdown of, the cell wall (such as expansion and pectinesterase). We speculate that these enzymes might contribute to supplying the carbon to the cytoplasm for MtLAP1-induced anthocyanin production. However, further work is needed to understand the role of these genes, if any, in MtLAP1-induced anthocyanin production.

### Infiltration-RNAseq in *Nicotiana benthamiana*

We have also utilised Infiltration-RNAseq via agroinfiltration of *N. benthamiana*. Here, we investigated three questions: (1) what are the transcriptional changes induced when *Agrobacterium* is infiltrated into a leaf, compared to infiltration of buffer only; (2) can Infiltration-RNAseq identify the targets of a TF from another plant species; (3) can the targets of a TF not normally expressed in leaves be identified using Infiltration-RNAseq.

Infiltration of *Agrobacterium* containing no construct into *N. benthamiana* leaves resulted in significant differential expression of a large number of genes (compared to buffer only; Additional file 7: Table S5). Upregulated genes included those involved in pathogenesis, and systemic acquired resistance (SAR) to overcome the perceived pathogen attack, such as pathogenesis-related (PR) proteins, WRKY transcription factors and disease resistance proteins. *Agrobacterium* infiltration resulted in the downregulation of chlorophyll binding proteins and photosynthetic metabolism genes. These transcriptional changes are likely the cause of the mild necrotic responses and leaf chlorosis symptoms elicited by disarmed *Agrobacterium* lab strains [48], which highlights the importance of using appropriate controls for assessing transcriptional changes via Infiltration-RNAseq (see “Methods”).

To determine if Infiltration-RNAseq can be used to identify the targets of a TF from another plant species, *N. benthamiana* leaves were agroinfiltrated with kiwifruit (*Actinidia chinensis*) *AcMYB10* [49], an anthocyanin regulator homologous to MtLAP1 and AtPAP1, and compared to infiltration with *Agrobacterium* containing no construct. This experiment identified anthocyanin biosynthetic genes and transcriptional regulators were significantly upregulated (Additional file 8: Table S6), which supports the findings from the Infiltration-RNAseq analysis of *Medicago* leaves infiltrated with *MtLAP1* (Fig. 3; Additional file 1: Table S1). In addition, it highlights that Infiltration-RNAseq can be employed in a range of plant species and targets of TFs from non-model plants can be identified in plants with optimised agroinfiltration protocols.

Our third experiment with *N. Benthamiana* was performed to ascertain whether Infiltration-RNAseq can identify the targets of a TF not normally expressed in leaves. To do this, we agroinfiltrated the *Arabidopsis thaliana* seed specific TF, *LEAFY COTYLEDON2* (*AtLEC2*) that plays a critical role during both early and late embryo development [50]; it acts to repress leaf traits and premature germination, and it is involved in the activation of seed storage proteins [51]. Compared to infiltration of *Agrobacterium* alone, agroinfiltration of *AtLEC2* resulted in the increased expression of seed storage proteins, including numerous oleosin proteins and 2S albumin storage protein, and various auxin-responsive genes (Additional file 9: Table S7). This result suggests that ectopic-expression of a TF via agroinfiltration can be used in conjunction with RNAseq to identify downstream target genes.

## Discussion

Agroinfiltration has been extensively used to study the biochemical function of proteins encoded by various plant genes; for example, it has been used to verify protein-protein interactions [8], identify protein subcellular localization [9], and to examine the ability of TFs to regulate promoter-reporter gene constructs [12]. However, because infiltration of *Agrobacterium* into a leaf will cause a large number of transcriptional changes, such as activation of genes involved in the plant’s immune response (Additional file 7: Table S5), it was unclear if this method could be used to reliably identify endogenous genes that might be regulated by the transient overexpression of specific TFs. Here, we show that by comparing the genes induced by *Agrobacterium* carrying a TF expressed under the control of the 35S promoter, with those induced by *Agrobacterium* carrying control constructs, we were able to identify TF-specific transcriptional changes.

*MtLAP1*, the MYB transcription factor that regulates the anthocyanin biosynthetic pathway in *Medicago* [25, 26], was used to investigate if the downstream targets of a TF could be identified using our Infiltration-RNAseq method. Our results showed that the complex regulation of the anthocyanin pathway was revealed in a single experiment where RNA sequencing data from *Medicago* leaves infiltrated with *MtLAP1* was compared to data obtained from control infiltrations. While studies using model plants have already provided a detailed understanding of the regulation of the anthocyanin pathway [37], identifying the corresponding genes in other plant species can be challenging. For example, in *Medicago*, there are more than 16 genes that potentially encode the enzyme Chalcone Synthase (CHS) (Additional file 10: Figure S3), which catalyses the first step in the biosynthesis of flavonoids. However, our results show that only two of these genes were strongly (approximately 30-fold) significantly upregulated by *MtLAP1* infiltration, while another three genes displayed weaker (approximately sixfold) significant upregulation (*P* value <0.05; Fig. 3; Additional file 1: Table S1). Similarly, *MtLAP1* infiltration caused the significant (*P* value <0.05) upregulation of other genes encoding enzymes in the anthocyanin pathway to varying degrees (approximately 8–40-fold) (Fig. 3; Additional file 1: Table S1). As well as identifying biosynthetic genes, genes associated with having higher levels of anthocyanins in the leaf, such as those encoding enzymes that modify or transport anthocyanins into the vacuole, were found to be upregulated (Additional file 1: Table S1). The spatiotemporal regulation of the anthocyanin pathway is precisely controlled by transcriptional activators and repressors from the MYB and basic helix-loop-helix (bHLH) protein families [52–54]. Our *Medicago* infiltration-RNAseq experiment identified MYBs and bHLH genes that were significantly upregulated by *MtLAP1* infiltration and are likely transcriptional activators and repressors of the anthocyanin pathway (Fig. 4; Additional file 1: Table S1). Of particular interest was the discovery of a *Medicago* R3-MYB, MtMYB730 (*Medtr2g088730*), which is likely to play a key role in the feedback repression of the MBW transcriptional activation complex. The observation that *MtMYB730* expression was activated by *MtLAP1* infiltration strongly suggests that feedback repression by R3-MYBs is also conserved in legumes; a feature of the anthocyanin gene regulation network that is proposed to occur throughout the eudicots [37], if not more widely [55].

The Infiltration-RNAseq method was further validated using *N. benthamiana*, a plant commonly used for agroinfiltration experiments. In this experiment, we compared the changes in gene expression caused by infiltrating *Agrobacterium* into *N. benthamiana* leaves with those caused by infiltrating buffer alone. As expected, the expression of a large number of genes, such as those encoding pathogenesis related (PR) proteins were significantly upregulated (Additional file 7: Table S5). As infiltration of *Agrobacterium* increases the expression of plant defence genes, we cannot rule out that this could mask the interpretation of results for TFs involved in defence. We envisage, for some defence-related TFs, the relevant changes in gene expression may not be seen, or the levels of gene expression change may not portray an accurate measure of how the TF effects expression of target genes. As such, we suggest caution should be taken when using Infiltration-RNAseq to understand the function of defence related TFs. This experiment also highlights the importance of using sufficient controls for an Infiltration-RNAseq experiment—we suggest using *Agrobacterium* either with or without a construct containing a transcriptional regulator that is independent of the TF being studied.

The use of an agroinfiltration control enabled TF-specific gene expression changes to be identified. Like the results obtained when *35S:MtLAP1* was infiltrated into *Medicago* leaves, the infiltration of the kiwifruit homologue of *MtLAP1*, *AcMYB10*, resulted in the significant upregulation of genes involved in anthocyanin biosynthesis and regulation in *N. benthamiana* (Additional file 8: Table S6). Similarly, infiltration of seed-specific *AtLEC2* resulted in genes normally only found in seeds, such as seed storage proteins (legumin, globulin, glutelin) and lipid binding or storage proteins (oleosins, glycine-rich lipid binding proteins, and Lipid transfer protein) being expressed in the infiltrated leaves (Additional file 9: Table S7). Recently, Grimberg et al., used a variation of Infiltration-RNAseq to identify transcriptional transitions in *N. benthamiana* leaves upon infiltration of five homologs of the TF *WRINKLED1*, which is involved in oil synthesis [14]. These experiments show that Infiltration-RNAseq has the power to determine the target genes of a TF of interest, even when they are not normally expressed in leaves.

Given the simplicity of Infiltration-RNAseq, this method could be used as a high-throughput way to analyse a large number of TFs. Currently, agroinfiltration is being used as a way to screen for TFs that might be involved in activating the expression of a particular gene or pathway [12]. This involves co-infiltrating leaves with *Agrobacterium* containing a promoter-reporter construct together with *Agrobacterium* containing the candidate TF construct. However, often multiple members of a TF family can activate a promoter-reporter gene construct making it difficult to know if the correct TF has been identified. Infiltration-RNAseq could be used to rapidly examine the *in planta* targets of a number of different TF family members. Our method would also be particularly valuable for studying TFs where a loss- or gain-of-function mutation induces an embryonic lethal phenotype.

Infiltration-RNAseq should be able to be used in any plant species where agroinfiltration protocols have been optimised, such as tomato [14], lettuce [19] potato [15], petunia [24] grapevine [16], grapefruit [17] and the medicinal plant *Maesa lanceolata* [18]. For many plants, such as *Medicago* and other legumes, making transgenic plants can be time-consuming and challenging. Infiltration-RNAseq provides another way to understand gene function and gene expression networks in this important family of plants that possess symbiotic nitrogen fixing capabilities.

## Conclusions

Infiltration-RNAseq provides a new and rapid way of identifying the targets of TFs. We have shown that this method works in both *Medicago* and *N. benthamiana*, that it can be used to identify TF target genes that are not normally expressed in leaves, and that targets of TFs from other plant species can be uncovered. This method should be applicable for any plant species in which transient agroinfiltration expression can be performed. It will be particular valuable for research in species, such as *Medicago*, lacking simple and reliable transformation protocols.

## Methods

### Plant methods

*Medicago truncatula* cv. R108 plants were scarified, germinated and grown as previously described [20]. *N. benthamiana* plants were grown as previously described [12].

### Agroinfiltration

For *Medicago* infiltrations, all three leaflets of the 4^th^ trifoliate leaf of healthy 3 weeks old plants were infiltrated with *Agrobacterium* (strain AGL-1) transformed with the *35S:MtLAP1* construct [25] or various control constructs (Fig. 1a). The control constructs include the following genes that are independent of the anthocyanin biosynthetic pathway and were being investigated as potential flowering time regulators [56, 57]: *Medtr7g018170* (*MtCOla*), *Medtr5g069480* (*MtCOlf*), *Medtr7g084970* (*MtFTa1*) and *Medtr5g022780* (*MtFD*). The coding sequences were sub-cloned into pCR8/GW/TOPO (Life technologies), checked by sequencing and then cloned into the Gateway compatible binary vector pB2GW7 [56–58]. This different set of experimental controls removes the effects of agroinfiltration i.e. genes involved in the pathogen disease response will be masked as they will be similarly expressed in leaves infiltrated with the control or test constructs. Preparation and delivery of the *Agrobacterium* suspension was performed as previously described [25].

For *N. benthamiana* infiltrations, preparation and delivery of the *Agrobacterium* suspension was performed as previously described [12, 27]. Briefly, plants were grown until they had six leaves and the youngest leaves over 1 cm long were infiltrated with Buffer, *Agrobacterium* (strain GV3101:pMP90), or *Agrobacterium* (strain GV3101:pMP90) transformed with *35S:AcMYB10* or *35S:AtLEC2* [59]. Each type of infiltration was performed in triplicate.

Optimisation of agroinfiltration will be required for different plant species and this will involve understanding when maximal expression of an infiltrated construct(s) occurs. Therefore, as with previous agroinfiltration experiments, we recommend sampling 3–4 days post infiltration and no later than about 5 days post infiltration as silencing of agroinfiltrated constructs is to be expected [12, 27].

### Transcriptome analysis of *Medicagotruncatula* agroinfiltrations

Infiltrated leaf tissue was harvested 4 days post infiltration with the intention to capture the highest gene expression changes of MtLAP1 target genes. Total RNA was extracted from the infiltrated leaf tissue using RNeasy Plant Mini Kit (Qiagen). RNAseq libraries were prepared from three control infiltrations (where one leaf was infiltrated with *35S:MtCOla*, *35S:MtCOlf* or *35S:MtFTa1plus35S:MtFD*) and three MtLAP1 infiltrations (where three leaves were infiltrated with *35S:MtLAP1* and named: 35S:MtLAP1_1, 35S:MtLAP1_2 and 35S:MtLAP1_3) using 500 ng of total RNA, and indexed with the TruSeq Stranded mRNA Library Prep Kit according to the protocol (Illumina). The same amount of each RNAseq library was pooled and run on one lane of HiSeq2000 100PE (Illumina).

The quality of the sequencing reads was assessed with FASTQC [60]. RNA sequencing reads were trimmed and filtered on length (≥25 bp) using Cleanadaptors [61]. Sequences (7–13 million reads per library) were aligned to the *M. truncatula* genome (version Mt4.0) using TopHat2 v2.0.14 [37], with default parameters and –library-type fr-firststrand, –b2-sensitive (version 2.2.6.0 of Bowtie2) (Additional file 11: Table S8). Visualisation and quantitation of the RNAseq libraries was performed with Seqmonk (v0.32.0) [62]. The RNAseq quantitation pipeline (within SeqMonk) was employed with uniquely mapped reads to generate a set of probes covering every mRNA in the genome, which were quantitated based on the number of reads falling within the exons of those mRNAs (ignoring any reads found in introns). The log-transformed counts produced were corrected for the total number of sequences in each dataset. The three control samples (35S:MtCOla, 35S:MtCOlf and 35S:FTa1plus35S:MtFD) and the three test samples (35S:LAP1_1, 35S:LAP1_2 and 35S:LAP1_3) were combined to make the ‘Control’ and ‘LAP1’ replicate sets, respectively (Fig. 2a; Additional file 12: Figure S4). Differential expression analysis between the two replicate sets was performed on raw counts with annotated mRNAs via the DESeq2 Filter (within SeqMonk) [35]. This output was combined with the Intensity Difference Filter (within SeqMonk), a statistical based fold change filter that works by constructing a local distribution of differences for mRNAs with similar average intensity to the mRNA being examined. Genes were considered significantly differentially expressed when the adjusted *P* value was <0.05 for both filters. In other words, the *P* value represents the likelihood that a given gene is significantly differentially expressed by chance, taking into consideration the absolute expression level and how variable other genes are that are expressed at a similar level. In general, highly expressed genes have low variability and lowly expressed genes have high variability. Hierarchical correlation clustering of the genes statistically differentially expressed between ‘Control’ and ‘LAP1’ was performed with SeqMonk (R-value threshold of 0.7). Differentially expressed genes were subjected to enriched GO categorization using the GO Analysis Toolkit and Agricultural Community (agriGO) database with default settings [44].

### Transcriptome analysis of *Nicotiana benthamiana* infiltrations

Infiltrated leaf tissue was harvested 3 days post infiltration. Total RNA was extracted from the infiltrated leaf tissue using RNeasy Plant Mini Kit (Qiagen). RNAseq libraries were prepared from the triplicate samples for each type of infiltration, using 500 ng of total RNA, and indexed with the TruSeq mRNA Library Prep Kit according to the protocol (Illumina). The same amount of each RNAseq library was pooled and run on one lane of HiSeq2000 100PE (Illumina).

 The quality of the sequencing reads was assessed with FASTQC [60]. RNA sequencing reads were trimmed using DynamicTrim (Phred score ≥20) and filtered on length (≥ 25 bp) using LengthSort [63]. RNAseq reads were aligned against the *N. benthamiana* transcriptome [64] using Bowtie2 v2.1.0 [65], and RSEM v1.2.3 [66] generated raw read counts for each transcript. DESeq [67] was used to run three differential expression analysis tests: (1) between Buffer infiltrated and *Agrobacterium* infiltrated; (2) between *Agrobacterium* and *Agrobacterium* containing *AcMYB10*; (3) between *Agrobacterium* and *Agrobacterium* containing *AtLEC2*. Lists of differentially expressed transcripts with a FDR adjusted *P* value <0.001 are shown in Additional file 7: Table S5, Additional file 8: Table S6, and Additional file 9: Table S7, respectively.

## Additional files

**Additional file 1: Table S1.** List of significanly differentially expressed *Medicago truncatula* genes between Control and LAP1 replicate sets (*P*-value <0.05).

**Additional file 2: Table S2.** Significantly differentially expressed *Medicago truncatula* genes (*P*-value <0.05) between Control and LAP1 replicate sets, falling into hierarchical cluster 1.

**Additional file 3: Table S3.** Significantly differentially expressed *Medicago truncatula * genes (*P*-value <0.05) between Control and LAP1 replicate sets, falling into hierarchical cluster 2.

**Additional file 4: Table S4.** Significantly differentially expressed *Medicago truncatula* genes (*P*-value <0.05) between Control and LAP1 replicate sets, falling into hierarchical cluster 3.

**Additional file 5: Figure S1.** *MtMATE2* (*Medtr1g100180*) up-regulation in response to agroinfiltration of MtLAP1.

**Additional file 6: Figure S2.** Maximum likelihood phylogenetic tree of *Medicago truncatula* MYB proteins.

**Additional file 7: Table S5.** Differentially expressed *Nicotiana benthamiana* transcripts between Buffer and *Agrobacterium* infiltrated replicate sets.

**Additional file 8: Table S6.** Differentially expressed *Nicotiana benthamiana* transcripts between infiltrated *Agrobacterium* and infiltrated *Agrobacterium* containing *AcMYB10* replicate sets.

**Additional file 9: Table S7.** Differentially expressed *Nicotiana benthamiana* transcripts between infiltrated *Agrobacterium* and infiltrated *Agrobacterium* containing *AtLEC2* replicate sets.

**Additional file 10: Figure S3.** Potential Chalcone Synthase genes in *Medicago truncatula*.

**Additional file 11: Table S8.** Summary of RNASeq alignments against the *Medicago truncatula* genome (Mt4.0).

**Additional file 12: Figure S4.** Scatter plots showing correlation between different samples in control and LAP1 replicate sets.

## Abbreviations

LAP1: *LEGUME ANTHOCYANIN PRODUCTION 1*
MYB: myeloblastosis
WDR: WD-repeat
bHLH: basic helix-loop-helix
MATE: multidrug and toxic compound extrusion
LEC2: LEAFY COTYLEDON2
TF: transcription factor
