# Supplementary material for: Infiltration-RNAseq: transcriptome profiling of Agrobacterium-mediated infiltration of transcription factors to discover gene function and expression networks in plants
Source: Plant Methods. 2016 Oct 19;12:41. doi: 10.1186/s13007-016-0141-7 (PMC5069895; doi:10.1186/s13007-016-0141-7)
Supplement: Supplementary file 5 — Additional file 5: Figure S1. MtMATE2 (Medtr1g100180) up-regulation in response to agroinfiltration of MtLAP1. [file 13007_2016_141_MOESM5_ESM.docx]

**
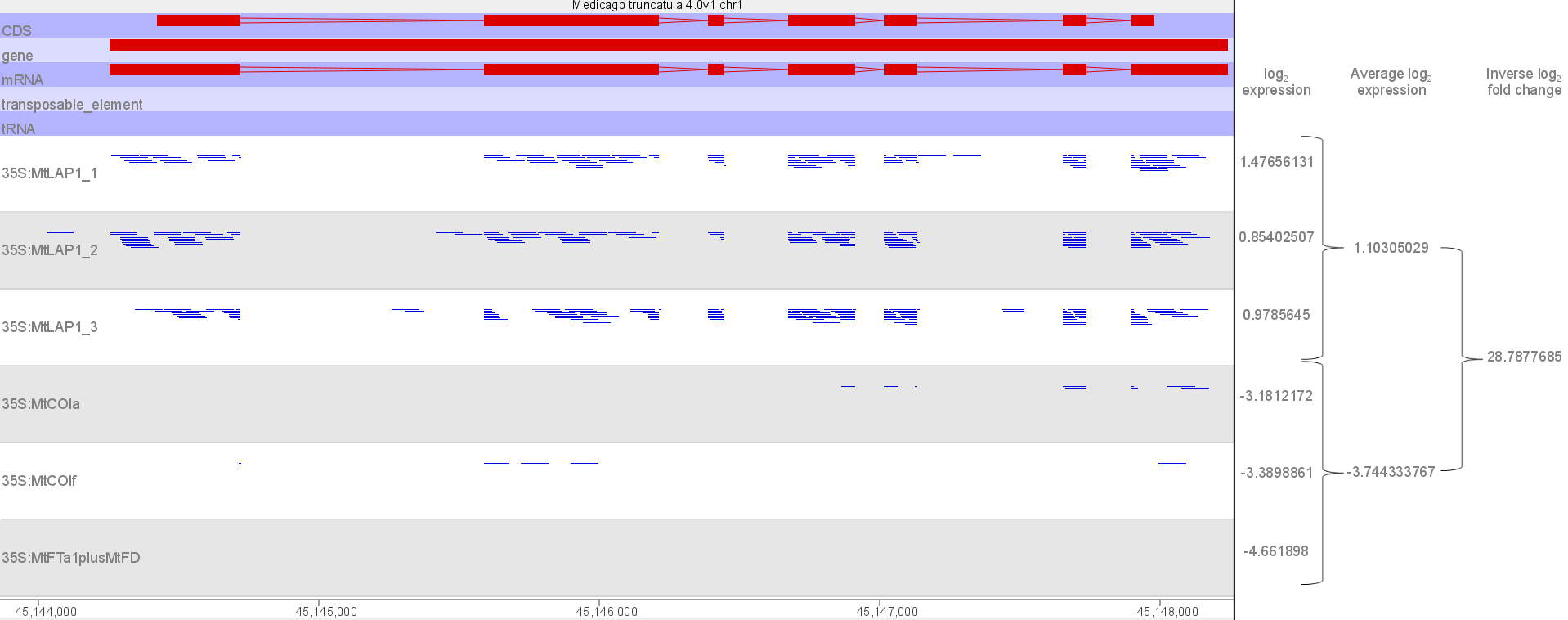
**

**Figure S1 *MtMATE2* (*Medtr1g100180*) up-regulation in response to agroinfiltration of *MtLAP1*.**

Snapshot of RNAseq reads mapping to *MtMATE2* (*Medtr1g100180* Chr1:45144255-45148243), visualised by SeqMonk [1]. The *MtMATE2* gene is on the forward strand (red) and the strand-specific RNAseq reads map to the reverse strand (blue). Log_2_ expression of *MtMATE2* is given for samples agroinfiltrated with *MtLAP1* (top three RNAseq tracks) and control constructs (bottom three RNAseq tracks), as determined by DESeq2 (*P*-value <0.05; [2]). *MtMATE2* is approximately 29-fold higher in *MtLAP1*-agroinfiltrated samples compared to control samples by this method. However, this did not hold true when the statistical-based fold change Intensity Difference Filter was incorporated (see methods), due to the large variation in expression between replicate samples.

1. Andrews S. SeqMonk [Internet]. [cited 2016 Apr 14]. Available from: http://www.bioinformatics.babraham.ac.uk/projects/seqmonk/

2. Love MI, Huber W, Anders S. Moderated estimation of fold change and dispersion for RNA-seq data with DESeq2. Genome Biol. 2014;15.
