# Supplementary material for: Infiltration-RNAseq: transcriptome profiling of Agrobacterium-mediated infiltration of transcription factors to discover gene function and expression networks in plants
Source: Plant Methods. 2016 Oct 19;12:41. doi: 10.1186/s13007-016-0141-7 (PMC5069895; doi:10.1186/s13007-016-0141-7)
Supplement: Supplementary file 6 — Additional file 6: Figure S2. Maximum likelihood phylogenetic tree of Medicago truncatula MYB proteins. [file 13007_2016_141_MOESM6_ESM.docx]

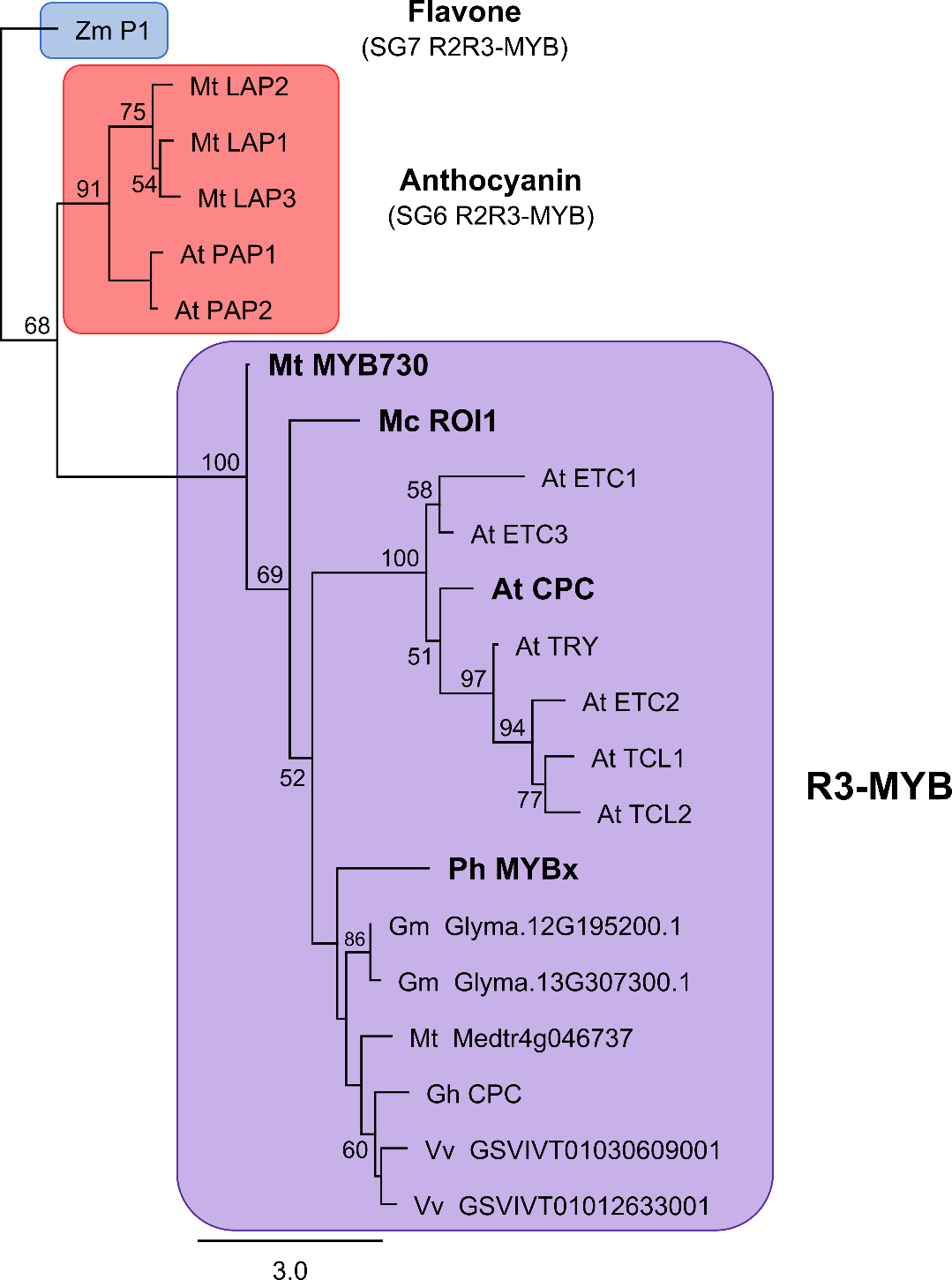


**Figure S2 Maximum likelihood phylogenetic tree of *Medicago truncatula* MYB proteins.**

R3-MYB proteins from *Arabidopsis thaliana* (At), *Glycine max* (Gm), *Gossypium hirsutum* (Gh), *Medicago truncatula* (Mt), *Mimulus cardinalis* (Mc), *Petunia hybrida* (Ph) and *Vitis vinifera* (Vv) were aligned and compared with R2R3-MYB proteins that regulate anthocyanin biosynthesis. The R2R3-MYB P1 from *Zea mays* (Zm) was used as an outgroup. Nodes with bootstrap support >50% are shown (1000 replicates). The R3-MYB proteins MYBx from *Petunia*, ROSE INTENSITY1 (ROI1) from *Mimulus* and CAPRICE (CPC) from *Arabidopsis* have established roles inhibiting MBW activity for anthocyanin pigmentation [1-3].

1. Zhu H-F, Fitzsimmons K, Khandelwal A, Kranz RG. CPC, a Single-Repeat R3 MYB, Is a Negative Regulator of Anthocyanin Biosynthesis in Arabidopsis. Mol. Plant. 2009;2:790–802.

2. Yuan Y-W, Sagawa JM, Young RC, Christensen BJ, Bradshaw HD. Genetic Dissection of a Major Anthocyanin QTL Contributing to Pollinator-Mediated Reproductive Isolation Between Sister Species of Mimulus. Genetics. 2013;194:255–63.

3. Albert NW, Davies KM, Lewis DH, Zhang H, Montefiori M, Brendolise C, et al. A conserved network of transcriptional activators and repressors regulates anthocyanin pigmentation in eudicots. Plant Cell. 2014;26:962–80.
