# Supplementary material for: Infiltration-RNAseq: transcriptome profiling of Agrobacterium-mediated infiltration of transcription factors to discover gene function and expression networks in plants
Source: Plant Methods. 2016 Oct 19;12:41. doi: 10.1186/s13007-016-0141-7 (PMC5069895; doi:10.1186/s13007-016-0141-7)
Supplement: Supplementary file 10 — Additional file 10: Figure S3. Potential Chalcone Synthase genes in Medicago truncatula. [file 13007_2016_141_MOESM10_ESM.docx]

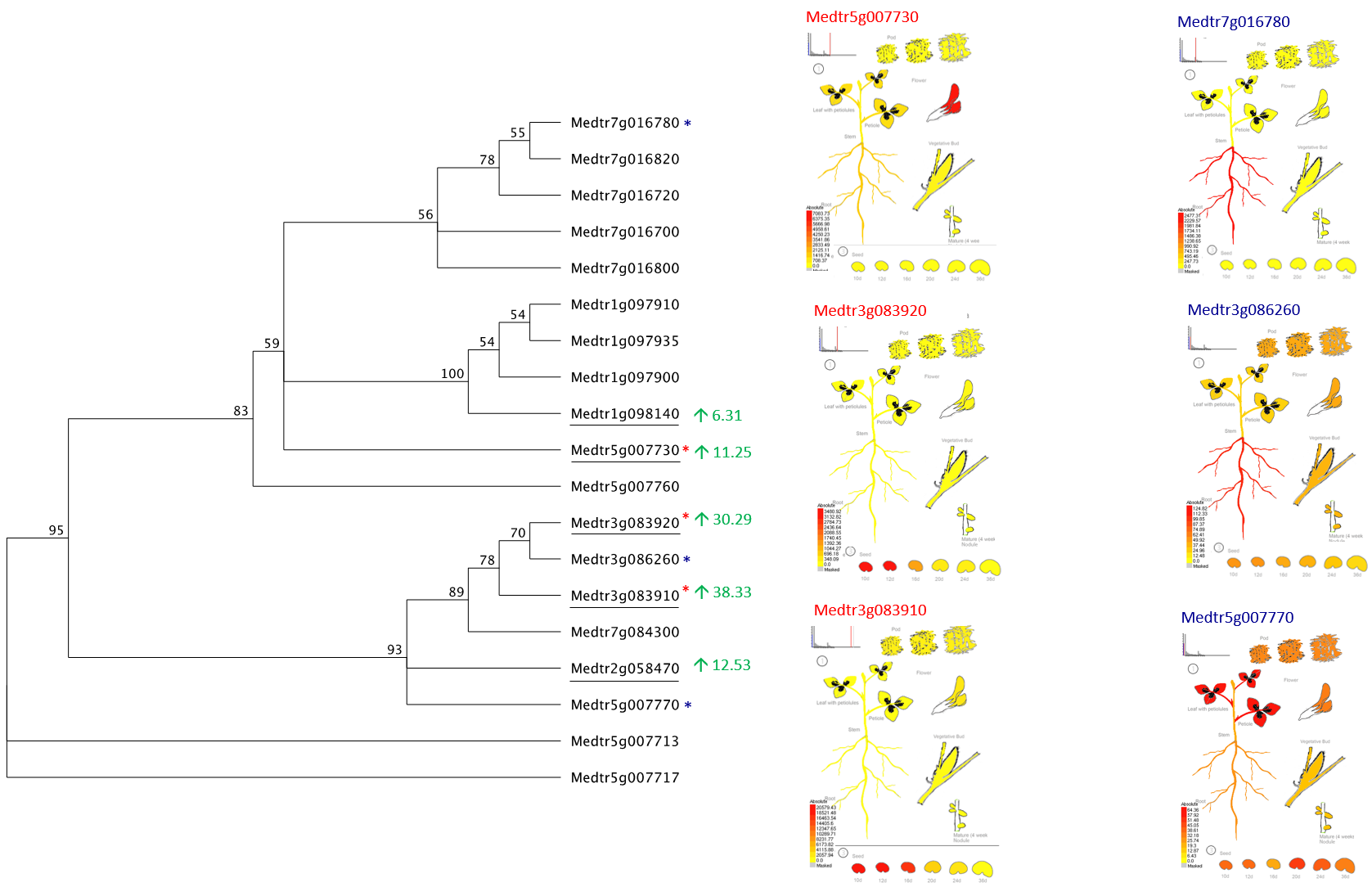


**Figure S3 Potential Chalcone Synthase genes in *Medicago truncatula.***

Phylogenetic tree of *Medicago truncatula* genes annotated as ‘Chalcone and Stilbene Synthase family protein’ (left). Bootstrap support for key branches is presented (%). Transcripts that are significantly differentially expressed (*P*-value <0.05) between Control and LAP1 conditions are underlined. Numbers in green are the fold change of expression observed for each significantly differentially expressed (*P*-value <0.05) gene between Control and LAP1 conditions (Table S1); green 🡩 represents upregulation. Potential Chalcone Synthase genes display different patterns of expression (right). Expression patterns of three genes (*Medtr5g007730*, *Medtr3g083920* and *Medtr3g083910*) that are significantly differentially expressed (*P*-value <0.05) between Control and LAP1 conditions (red *) are predominantly expressed in leaf, floral and seed tissue. In contrast, expression patterns of three other genes (*Medtr1g016780*, *Medtr3g086260* and *Medtr5g007770*), which are not differentially expressed between Control and LAP1 conditions (blue *), are predominately expressed in roots or show a more general expression pattern. The *Medicago* electronic fluorescence pictographic (eFP) browser [1–3], via the Bio-Analytic Resource (BAR) for Plant Biology [4], was used to investigate gene expression profiles of potential Chalcone Synthase genes.

1. Benedito VA, Torres-Jerez I, Murray JD, Andriankaja A, Allen S, Kakar K, et al. A gene expression atlas of the model legume Medicago truncatula. Plant J. 2008;55:504–13.

2. Patel R V, Nahal HK, Breit R, Provart NJ. BAR expressolog identification: expression profile similarity ranking of homologous genes in plant species. Plant J. 2012;71:1038–50.

3. Medicago eFP Browser [Internet]. [cited 2016 Jun 3]. Available from: http://bar.utoronto.ca/efpmedicago/cgi-bin/efpWeb.cgi

4. The Bio-Analytic Resource (BAR) for Plant Biology [Internet]. [cited 2016 Jun 3]. Available from: http://bar.utoronto.ca/
