# Supplementary material for: Infiltration-RNAseq: transcriptome profiling of Agrobacterium-mediated infiltration of transcription factors to discover gene function and expression networks in plants
Source: Plant Methods. 2016 Oct 19;12:41. doi: 10.1186/s13007-016-0141-7 (PMC5069895; doi:10.1186/s13007-016-0141-7)
Supplement: Supplementary file 11 — Additional file 11: Table S8. Summary of RNASeq alignments against the Medicago truncatula genome (Mt4.0). [file 13007_2016_141_MOESM11_ESM.docx]

**Table S8 Summary of RNASeq alignments.**

| **Library** | | | **Alignment rate** | **# read pairs mapped** |
| --- | --- | --- | --- | --- |
| 1 | 35S:MtLap1_1 | LAP1 | 73.7% | 9922523 |
| 2 | 35S:MtLap1_2 | LAP1 | 73.9% | 7717499 |
| 3 | 35S:MtLap1_3 | LAP1 | 73.3% | 5696422 |
| 4 | 35S:MtCOla | Control | 74.4% | 7620056 |
| 5 | 35S:MtCOlf | Control | 75.4% | 8200843 |
| 6 | 35S:MtFTa1plusMtFD | Control | 75.4% | 8200843 |
