# Supplementary material for: Infiltration-RNAseq: transcriptome profiling of Agrobacterium-mediated infiltration of transcription factors to discover gene function and expression networks in plants
Source: Plant Methods. 2016 Oct 19;12:41. doi: 10.1186/s13007-016-0141-7 (PMC5069895; doi:10.1186/s13007-016-0141-7)
Supplement: Supplementary file 12 — Additional file 12: Figure S4. Scatter plots showing correlation between different samples in control and LAP1 replicate sets. [file 13007_2016_141_MOESM12_ESM.docx]

**
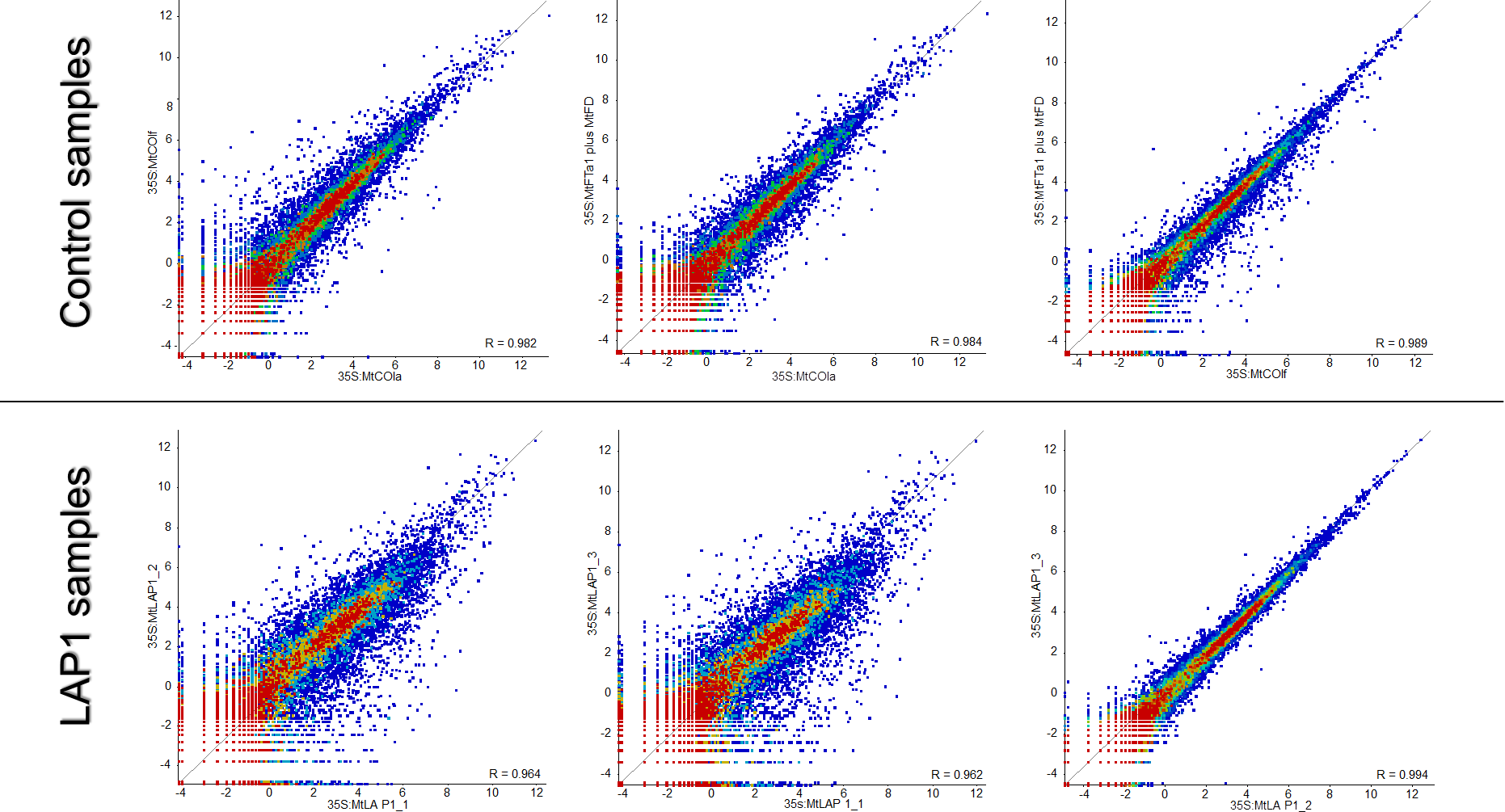
**

**Figure S4 Scatter plots showing correlation between different samples in Control and LAP1 replicate sets.**

Each individual point on the different plots represents a probe (an individual *Medicago truncatula* mRNA), showing the relationship between the quantitated values in the two selected data stores (as stated on the x- and y-axis of each scatter plot). The Pearson correlation value for each test is given within each plot.
